# Supplementary material for: Maintenance of appropriate size scaling of the C. elegans pharynx by YAP-1
Source: Nat Commun. 2023 Nov 20;14:7564. doi: 10.1038/s41467-023-43230-1 (PMC10661912; doi:10.1038/s41467-023-43230-1)
Supplement: Supplementary file 1 — Supplementary Information [file 41467_2023_43230_MOESM1_ESM.pdf]

Maintenance of appropriate size scaling of the *C. elegans* pharynx by YAP-1

Klement Stojanovski[1], Ioana Gheorghe[1], Peter Lenart[1], Anne Lanjuin[2], William B. Mair[2], Benjamin D. Towbin[1,3]

[1] University of Bern, Bern, Switzerland.

[2] Dept. Molecular Metabolism, Harvard TH Chan School of Public Health

[3] to whom correspondence should be addressed: benjamin.towbin@unibe.ch

Supplemental Information:

- Supplemental Figures 1-9

- Supplemental Tables 1-4

Supplemental Figures

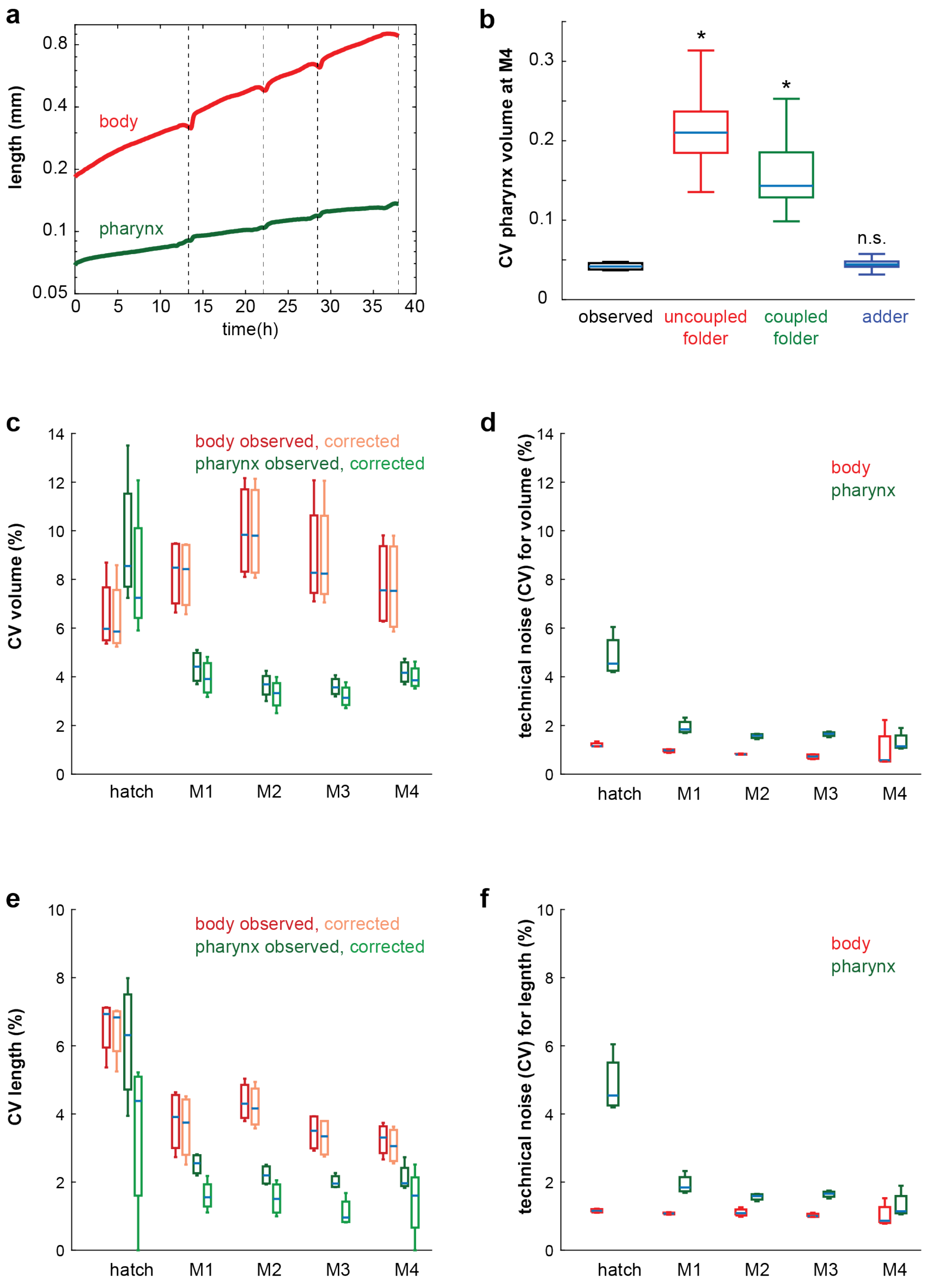

Supplemental Figure 1. Pharyngeal length is less heterogeneous among individuals than total body length

a) Body (red) and pharynx (green) length as a function of time averaged from n=475 individuals. For averaging, individuals of each larval stage were re-scaled to have matching larval stage entry and exit points and the growth curve was scaled back to the mean larval stage duration.

b) Comparison of observed CV of pharyngeal volume to randomized simulations at indicated larval moults. *p = 0.029 (ranksum test simulated vs observed), n.s. p = 0.34
c) Coefficient of variation (CV) of body (red) and pharynx (green) length at hatch and larval moults. Lighter colors indicate CV corrected for technical measurement noise. Box plots represent CVs of 4 independent day-to-day repeats with $105 < n < 181$ individuals per repeat.
d) Estimation of technical measurement noise at indicated stages from linear regression to 10 time points immediately prior to moults or immediately after hatching.
e) Same as c., but for body length
f) Same as d., but for body length

Boxplots: central line: median, box: interquartile ranges (IQR), whisker: ranges except extreme outliers (>1.5*IQR)

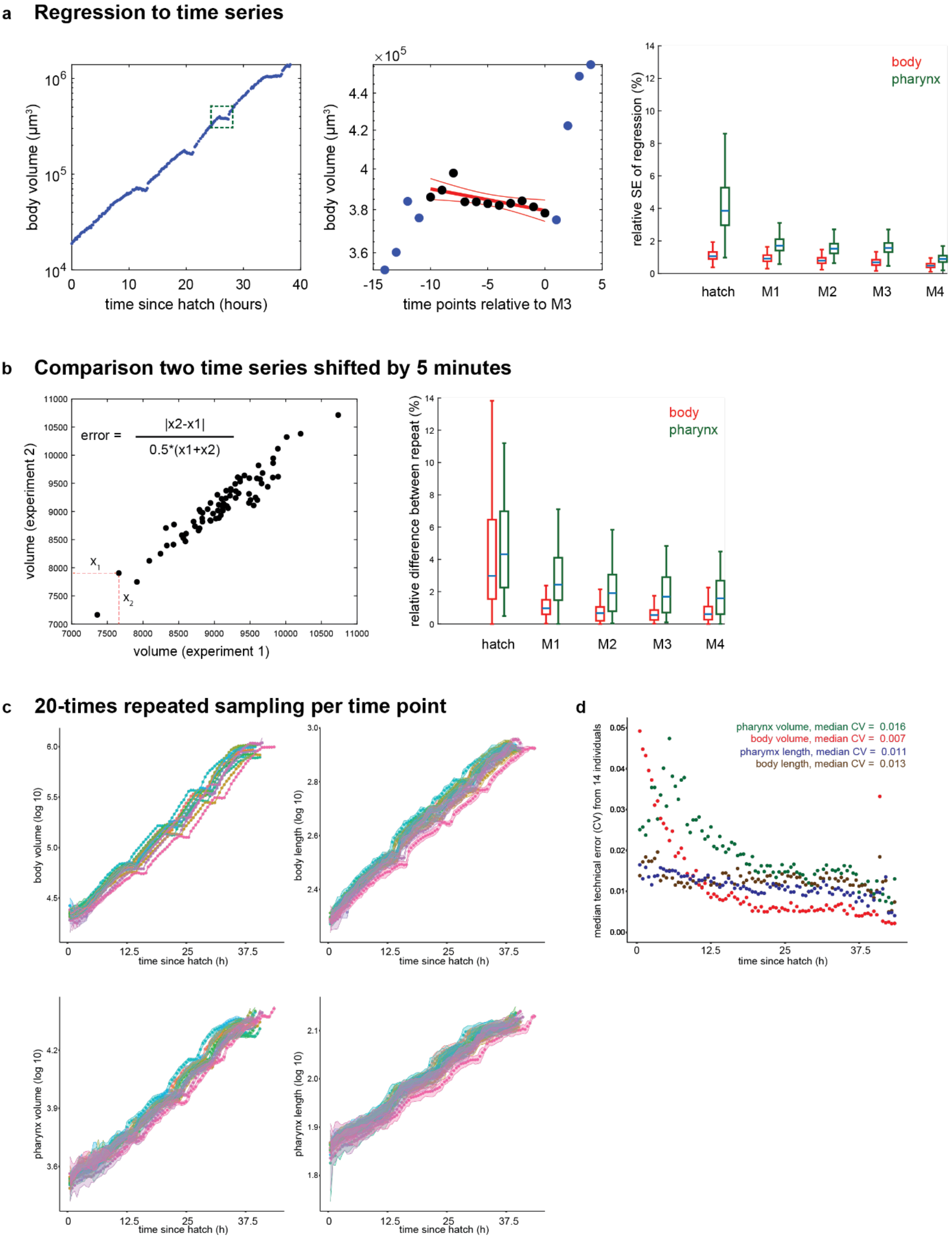

Supplemental Figure 2. Estimation of measurement noise

a) Illustration of method used to determine volumes at molts, including measurement error by linear regression. For analyses in Figure 1 and 2, volumes at molts were determined by a linear regression to log (volume) to the 10 timepoint prior to the molt. Volume at hatch was

determined by a linear regression to log (volume) to the first 10 time points after hatching. The standard error of the linear regression informs on the measurement error. Left: body volume trajectory of a single individual from hatch to 100 minutes after M4. Data in green square is magnified in the middle panel. blue circles: measurement points, black circles: measurement points used for linear regression, red: linear regression +/- 95% confidence interval. Box plot in the right panel shows the relative standard error of the linear regression for pharynx and body. central line: median, box: interquartile ranges (IQR), whisker: ranges except extreme outliers (>1.5*IQR)

b) Correlation of body volume for the same individual between experimental repeats. Volume growth trajectories of n = 60 individuals were measured in micro chambers at a 5 minute time resolution. Time series was split into two separate datasets of 10 minute time resolution, shifted relative to each other by 5 minutes. Volumes at hatch and molts were then determined separately for each dataset by the same method as for data shown in Figures 1 and 2. Scatter plot on the left shows the correlation between the two measurements of volume at M3 for each individual. Box plots on the right show the mean relative difference between the two repeated measurements for body (red) and pharynx (green). central line: median, box: interquartile ranges (IQR), whisker: ranges except extreme outliers (>1.5*IQR)

c) Measurement error (CV) of pharynx and body length and volume at individual timepoints (prior interpolation by regression). n = 14 individuals were imaged in micro chamers at 30 min interval and m = 20 consecutive images were acquired at each time point. A separate auto-focus performed for each acquisition. Plots show measurements of the 14 individuals. Circles indicate mean of 20 repeated measurements. Shaded area is +/-1.96*SD among the 20 repeated measurements. When the shaded area is invisible, the error is smaller than the marker.

d) Median measurement error for individual time points across n=14 individuals shown in c) as a function of time since hatch. The measurement error for each individual and time point was computed as the coefficient of variation (CV_{indiv}) of the 20 measurements. Circles are the median values of the CV_{indiv} at each time point.

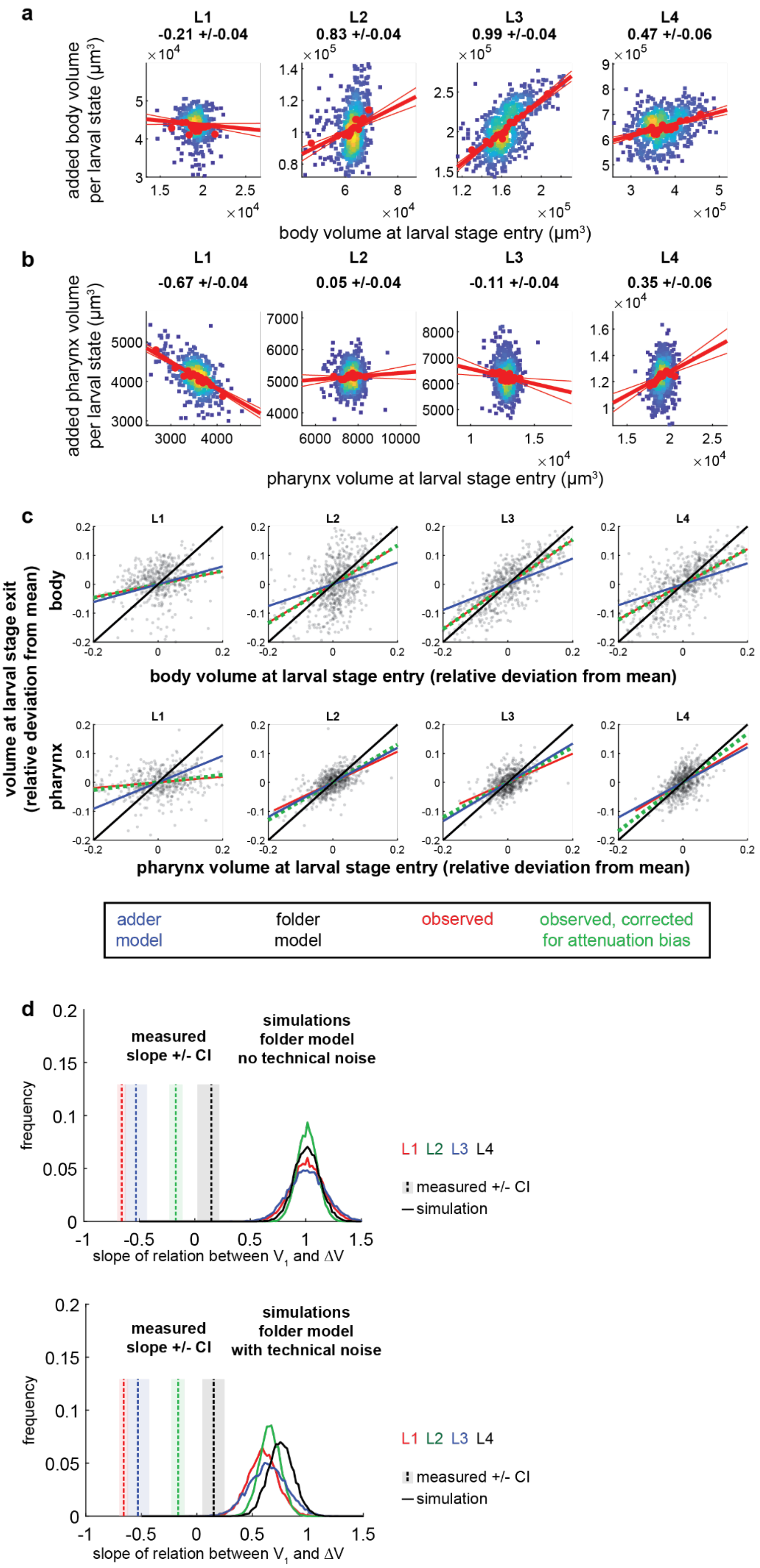
a
L1
-0.21 +/-0.04
L2
0.83 +/-0.04
L3
0.99 +/-0.04
L4
0.47 +/-0.06
added body volume per larval state (μm³)
body volume at larval stage entry (μm³)
b
L1
-0.67 +/-0.04
L2
0.05 +/-0.04
L3
-0.11 +/-0.04
L4
0.35 +/-0.06
added pharynx volume per larval state (μm³)
pharynx volume at larval stage entry (μm³)
c
volume at larval stage exit (relative deviation from mean)
body
body volume at larval stage entry (relative deviation from mean)
pharynx
pharynx volume at larval stage entry (relative deviation from mean)
adder model
folder model
observed
observed, corrected for attenuation bias
d
measured slope +/- CI
simulations folder model no technical noise
frequency
slope of relation between V1 and ΔV
L1 L2 L3 L4
measured +/- CI
simulation
measured slope +/- CI
simulations folder model with technical noise
frequency
slope of relation between V1 and ΔV
L1 L2 L3 L4
measured +/- CI
simulation

Supplemental Figure 3. Linear pharyngeal volume growth within larval stages produces an adder-like behaviour

a) Scatter plot of pharynx volume at larval stage entry vs. added pharynx volume per larval stage for individuals. Numbers above chart indicate slope of regression line +/- 95% CI. Red circles: binned average along x-Axis. red line: robust linear regression (thick) with confidence intervals (thin). n = 475,641, 641, 638 individuals for L1 to L4 from 4 day-to-day repeats.

b) As a., but for pharynx volume

c) Same data from measurements in Figures 2a-b, but displayed as volume at beginning of larval stage (V_1) vs. volume at end of larval stage (V_2) instead of V_1 vs. ΔV to allow for correction of attenuation bias. Red: linear regression to observed data, green: linear regression corrected for attenuation bias, blue: slope expected for adder, black: slope expected for folder. Note that the expected slope for an adder differs between larval stages, since the slope between mean normalized V_1 and V_2 of an adder depends on the volume fold change undergone in this stage [$slope_{adder}$ = 1/(fold change)].

d) Outcome of simulation of a folder model with and without technical noise vs. measured data. Histograms (solid lines) show the slopes between V_1 and ΔV for different larval stages produced by 10'000 simulations of a folder model with the same number of individuals as was measured experimentally. For each simulation, starting volumes were drawn from a normal distribution with CV matching the measured data (corrected for technical error) and multiplied with fold changes drawn from a normal distribution with CV matching measured data. Subsequently, for the bottom graph, technical noise was added, drawn from a normal distribution with CV matching the experimentally determined technical noise. For each simulation, the slope was determined from a linear regression to V_1 vs. ΔV, normalized to their respective means, and the distribution of slopes from individual simulation outcomes is shown as a histogram. Without technical noise, the histogram is centred around 1. Addition of technical noise shifts the histogram to lower values due to attenuation bias. Dotted lines show the experimentally measured relation between V_1 and ΔV as shown in Figure 2b +/- 95% CI (shaded area). The experimentally observed slopes are smaller than the slope produced by each of the 10'000 simulations, such that a folder model can be rejected.

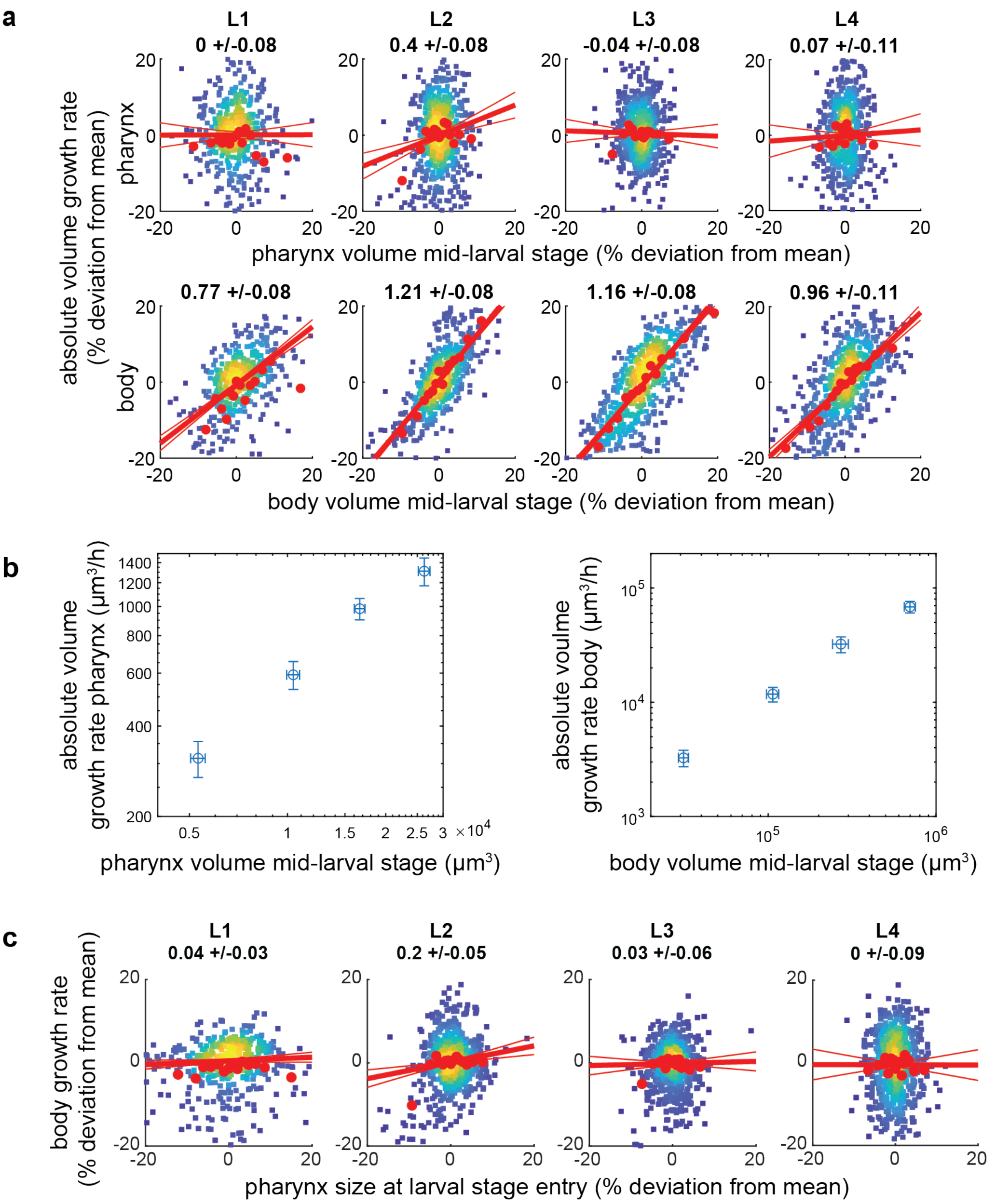

Supplemental Figure 4. The pharynx grows linearly within larval stages, but exponentially across larval stages

a) Scatter plot of pharynx volume (top) and body volume (bottom) at 40% of the larval stage vs. Absolute rate of pharynx and body volume increase. Data was normalized to batch mean. Outliers outside of the +/- 20% range are omitted for better visualization. Numbers above chart indicate slope of regression line +/- 95% CI. Red circles: binned average along x-Axis. Red line: robust linear regression (thick) with confidence intervals (thin). n = 475,641, 641, 638 individuals for L1 to L4 from 4 day-to-day repeats.

b) Mean pharyngeal and body volume at 40% of the larval stage (L1 to L4 from left to right) vs. absolute rate of volume increase. Error bars: standard deviation. Near linear scaling of growth and

size in a log-log plot is consistent with near exponential growth across larval stages. Number of individuals is the same as for a).

c) As a), but for pharynx volume at larval stage entry vs. body volume growth rate (Δlog(V)/Δt) in the same larval stage.

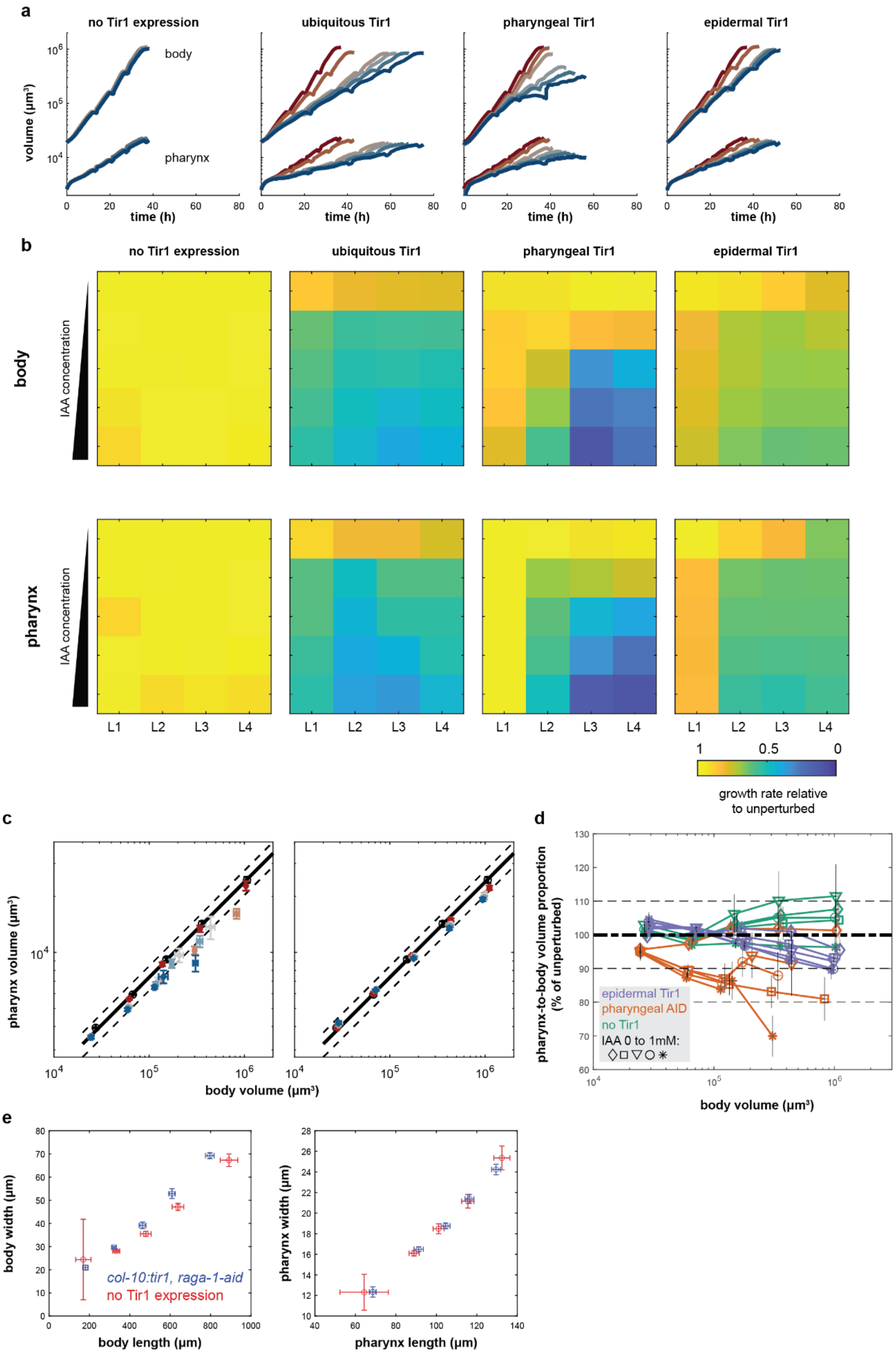

Supplemental Figure 5. Pharynx-to-body volume proportions are robust to tissue-specific depletion of RAGA-1

a) Body (top) and pharynx (bottom) volume as a function of time with depletion of RAGA-1 in indicated tissues. Color indicates IAA concentration from red to blue: no Tir1 expression 0mM IAA, Tir1 expression + 0mM, 0.1mM, 0.25mM, 0.5mM, 1mM IAA. Number of individuals n, $26 < n < 259$ from number of day-to-day repeats m, $3 < m < 11$. See Supplemental Tables 1 and 2 for n and m of each condition.

b) Heatmap showing volume growth rate of body and pharynx normalized to unperturbed growth (no Tir expression and no IAA) after RAGA-1 depletion by AID in indicated tissues, larval stages, and IAA concentrations. IAA concentrations increase from top to bottom as follows: 0mM, 0.1mM, 0.25mM, 0.5mM, 1mM

c) Scatter plot showing body vs. pharynx volume at the beginning of L1 and at all larval moults M1 to M4 (circles in order from left to right) under pharyngeal (left) or epidermal (right) AID of RAGA-1. black circles: relation between pharynx and body length when unperturbed (no Tir expression and no IAA). Solid black line: linear regression to unperturbed body-to-pharynx length (P-line). Dashed black line: 15% deviation from P-line. Coloured circles: IAA concentrations from red to blue: 0, 100, 250, 500, 1000µM. Error bars are standard error of the mean among day-to-day repeats. Slope m of P-line for IAA concentrations from 0 to µM: pharyngeal RAGA-1 AID: 0.49, 0.43, 0.48, 0.46, 0.37; epidermal RAGA-1 AID: 0.47, 0.45, 0.45, 0.44, 0.44; no Tir expression: 0.50, 0.49, 0.50, 0.50, 0.47

d) Deviation of pharynx-to-body volume ratio from the unperturbed P-line vs. body volume at early L1 and larval moults. Colours indicate different Tir1 expression; orange: pharynx, purple: epidermis, green: no Tir1 expression. Symbols indicate IAA concentration from 0mM (diamond) to 1mM (asterisk). Error bars are standard errors among day-to-day repeats. Where invisible, error bars are smaller than the marker.

e) Body and pharynx length vs. width for *col10p:tir1; raga-1-aid* (blue) and for *raga-1-aid* without Tir1 expression (red) at 1mM IAA at hatch and all four moults from left to right. Error bars are the standard deviation among individual animals.

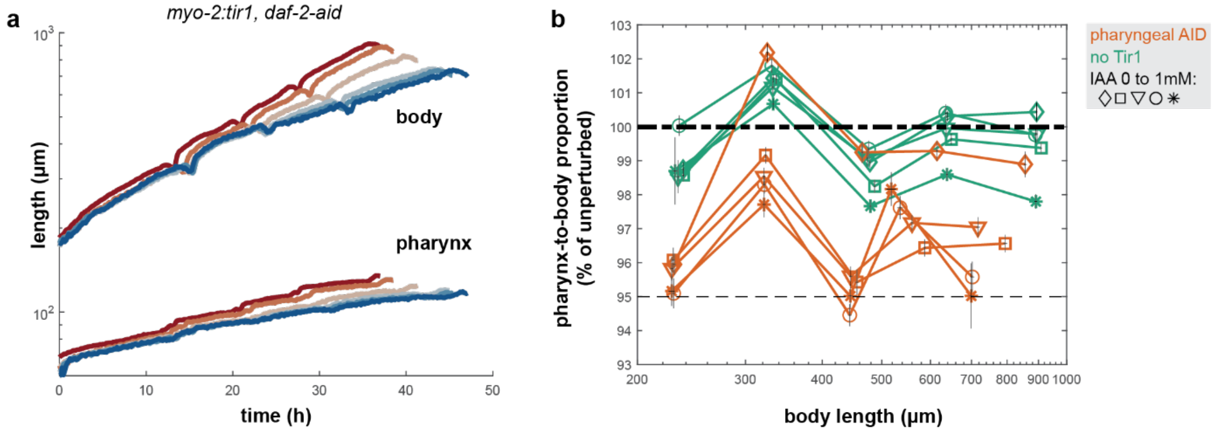

Supplemental Figure 6. Pharynx-to-body volume proportions are robust to tissue-specific depletion of DAF-2

a) Body (top) and pharynx (bottom) length as a function of time with depletion of DAF-2 in the pharynx. Color indicates IAA concentration from red to blue as follows: no Tir1 expression 0mM IAA, Tir1 expression + 0mM, 0.1mM, 0.25mM, 0.5mM, 1mM IAA.

b) Deviation from unperturbed pharynx-to-body length ratio (deviation from P-line) vs. body length in early L1 and at larval moults upon DAF-2 AID in the pharynx (orange) and without DAF-2 AID (green). Symbols indicates IAA concentration from 0mM (diamond) to 1mM (asterisk). Error bars are standard errors among day-to-day repeats. Thick dashed lines indicate unperturbed state (P-line). Thin dashed line indicates a 5% deviation from te P-line.

a), b). 17 < n <58 individuals from 2 day-to-day repeats. See Supplemental Tables 1 and 2 for exact number of individuals for each condition.

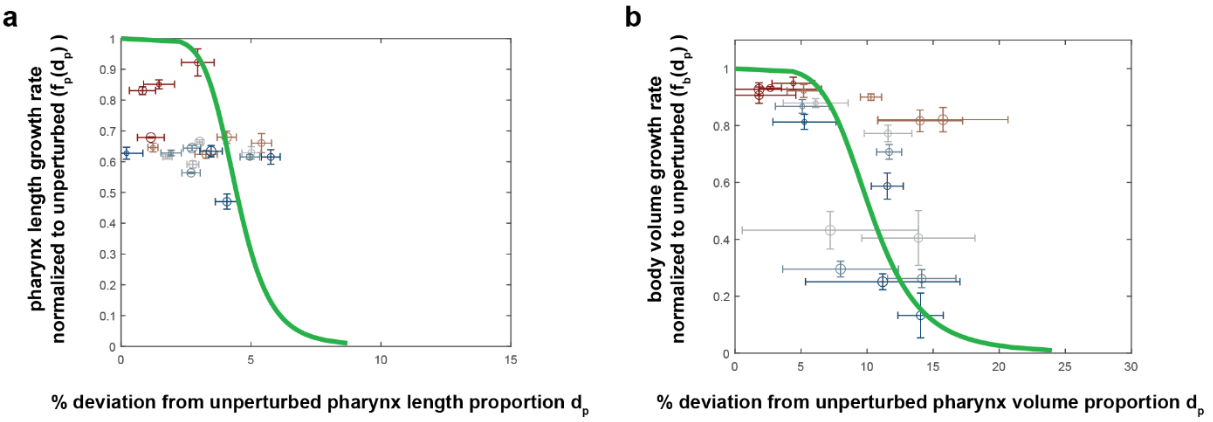

Supplemental Figure 7. An ultra-sensitive relation between pharynx size and body growth is required for robustness of pharynx-to-body length proportions to tissue-specific growth inhibition

a) Pharynx length growth rate as a function of deviation of pharynx length from the P-line under epidermal growth inhibition normalized to unperturbed growth. Colour indicates IAA concentration increasing from red (0 mM) to blue (1 mM). Circle size indicates the larval stage. Green line is the Hill function obtained from fit to the pharyngeal RAGA-1 AID described in the main text and shown in Figure 4a.

b) Body volume growth rate as a function of deviation of pharynx volume from the P-line under pharyngeal growth inhibition normalized to unperturbed growth. Colour indicates IAA concentration increasing from red (0 mM) to blue (1 mM). Circle size indicates the larval stage. Green line is a fitted Hill function as described in the main text.

a), b). error bars indicate standard error of the mean between day-to-day repeats.

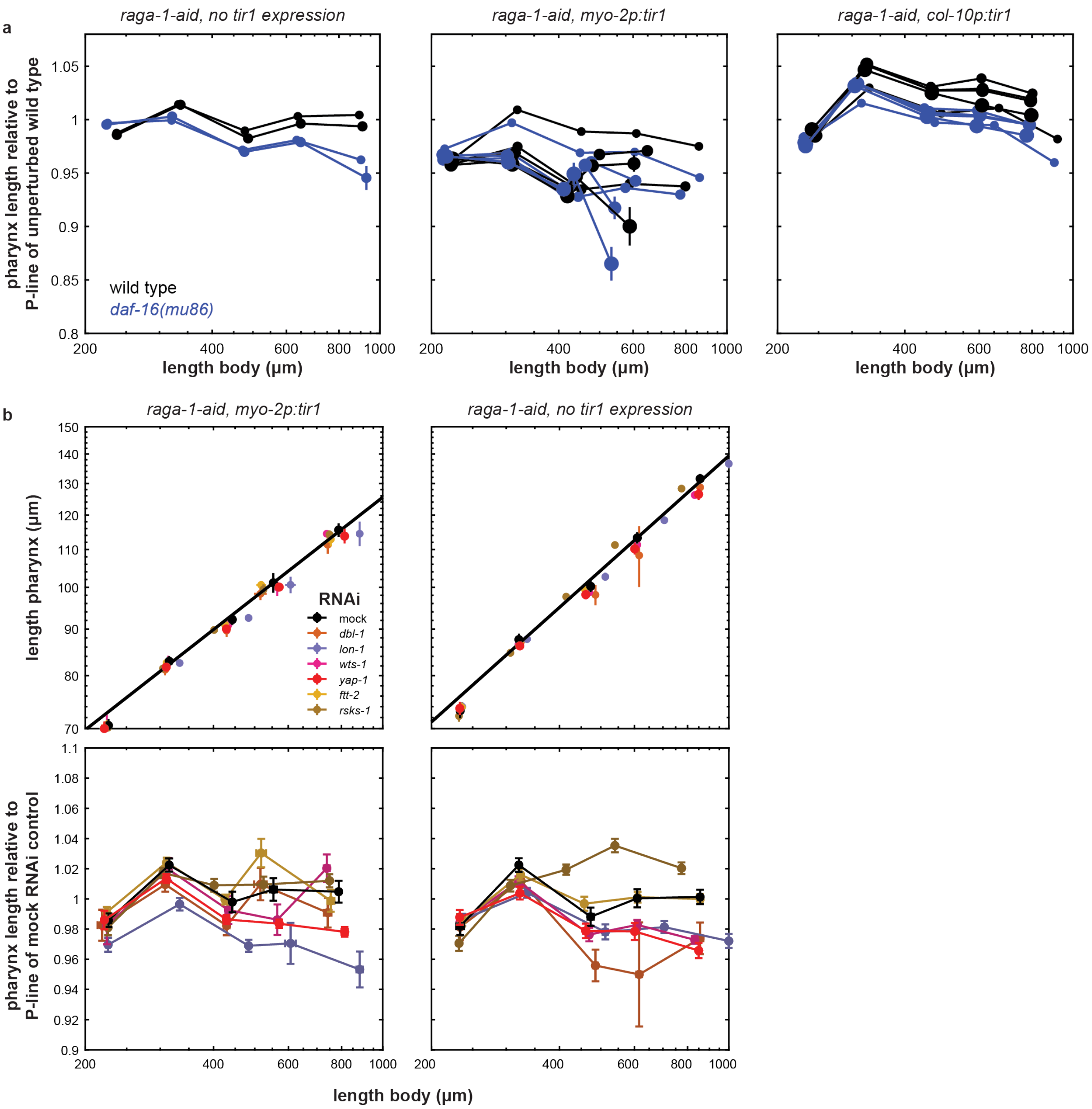

Supplemental Figure 8. *yap-1* is required for robustness of pharynx-to-body volume proportions to epidermal growth inhibition

a) Deviation of pharyngeal length of *daf-16(mu86)* mutants from P-line fitted to the unperturbed wild type. Connected circles indicate 30% L1, and M1 to M4 moults (left to right) of indicated genotypes and RAGA-1 AID treatments. IAA concentrations: without Tir1 expression (left): 0mM, 0.1mM. For pharyngeal (middle) and epidermal (right) Tir1 expression: 0mM, 0.1mM, 0.25mM, 0.5mM, 1mM. Marker size indicates IAA concentration (smallest marker for lowest concentration). Error bars are standard error of the mean among individuals. See Supplemental Tables 1 and 2 for number of individuals and day-to-day repeats of each condition.

b) Top: body length vs. pharynx length for *raga-1-aid* strain with Tir1 expression in the pharynx (left) or without Tir1 expression (right) for indicated RNAi. Bottom: body length vs. deviation of pharyngeal length from P-line fitted to the mock RNAi control for indicated RNAi. IAA concentration: 0.1mM. error bars are standard error of the mean among individuals.

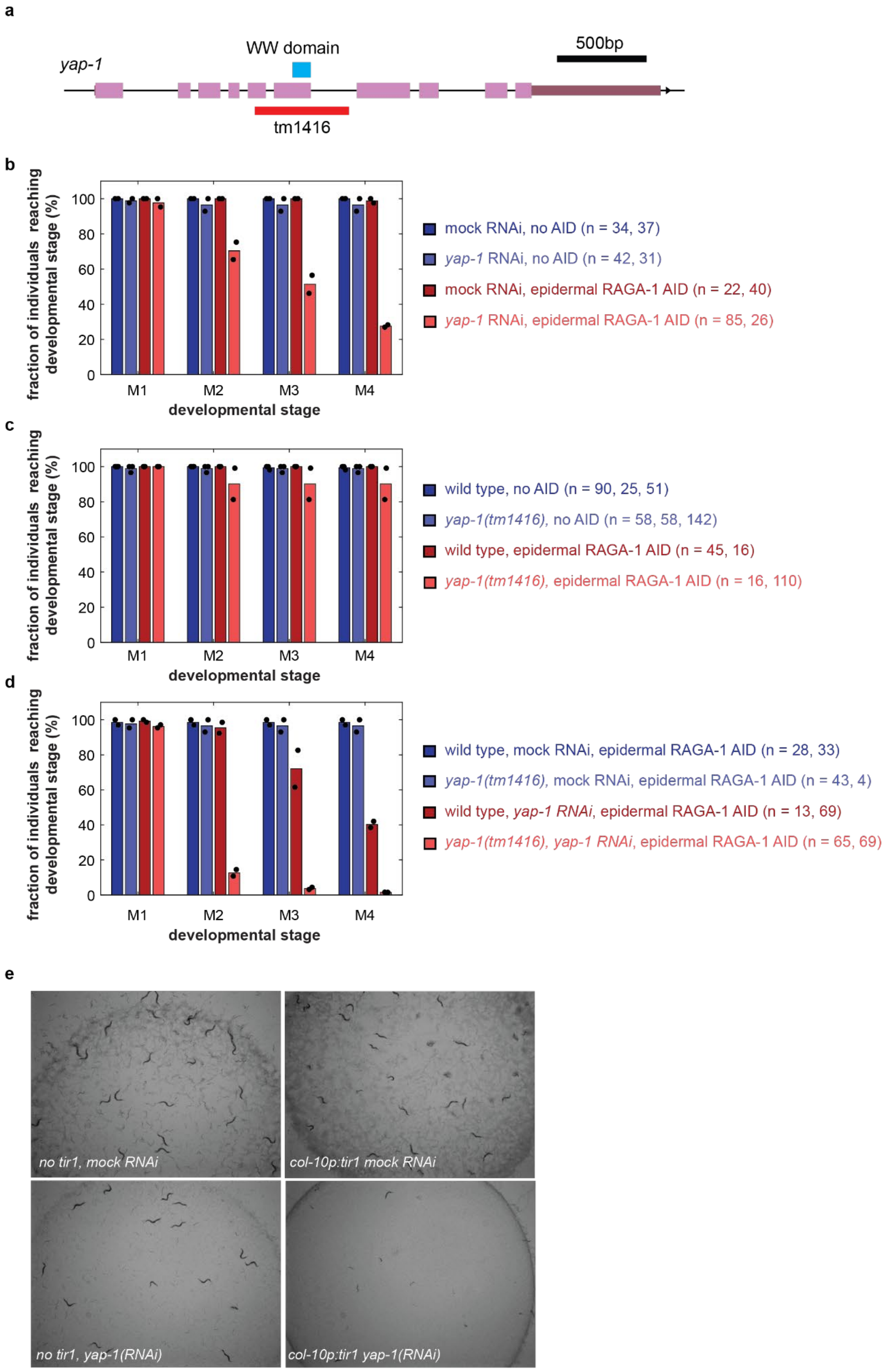

Supplemental Figure 9. ***yap-(RNAi)*** **causes larval arrest when combined with epidermal RAGA-1 AID**

a) exon structure of *yap-1* locus and location of *tm1416* deletion allele relative to the WW domain. Pink: exons, brown: untranslated region.

b) Fraction of animals reaching M1, M2, M3, and M4 for indicated treatment (with and without epidermal RAGA-1 AID, and with and without *yap-1(RNAi)* grown in micro chambers. Bars are mean of 2 day-to-day repeats. n indicates number of individuals per repeat. Black dots indicate values for individual days.

c) As b) but for wild type and *yap-1(tm1416)* instead of *yap-1(RNAi)* and three day-to-day repeats for no AID controls.

d) As for c), but *yap-1(RNAi)* was applied to wild type and *yap-1(tm1416)* animals. All groups were treated with epidermal RAGA-1 AID.

e) Images of animals grown on RNAi plates under indicated conditions. RNAi was initiated from the L4 stage of the parental generation. Animals were synchronized at L1 stage by bleaching and grown on RNAi plates containing 0.5mM IAA for 72 hours. *yap-1(RNAi)* animals also arrest in development when treated with epidermal RAGA-1 AID on plates instead of micro chambers.

Supplemental Table 1. number of day-to-day repeats per condition

IAA	no Tir1 expression, *raga-1-aid*	*eft-3p:tir1, raga-1-aid*
0mM	10	4
0.1mM	10	4
0.25mM	7	4
0.5mM	7	4
1mM	7	4

IAA	*myo-2p:tir1, raga-1-aid*	*col-10p:tir1, raga-1-aid*
0mM	8	4
0.1mM	8	4
0.25mM	5	4
0.5mM	5	4
1mM	5	4

IAA	*myo-2p:tir-1, daf-2-aid*	*myo-2p:tir1, raga-1-aid, daf-16(mu86)*
0mM	2	5
0.1mM	2	5
0.25mM	2	2
0.5mM	2	2
1mM	2	2

IAA	*col-10p:tir1, raga-1-aid, daf-16(mu86)*	no Tir1 expression, *raga-1-aid* , *daf-16(mu86)*
0mM	2	2
0.1mM	2	2
0.25mM	2	na
0.5mM	2	na
1mM	2	na

**Supplemental Table 2. total number of individuals tested**				
no Tir1 expression, *raga-1-aid*				
IAA	L1	L2	L3	L4
0mM	142	244	242	220
0.1mM	104	171	171	153
0.25mM	53	107	106	98
0.5mM	61	98	98	89
1mM	83	123	120	99
eft-3p:tir1, raga-1-aid				
IAA	L1	L2	L3	L4
0mM	52	68	68	66
0.1mM	53	86	86	84
0.25mM	52	71	69	61
0.5mM	50	75	72	58
1mM	55	80	71	57
myo-2p:tir1, raga-1-aid				
IAA	L1	L2	L3	L4
0mM	159	258	258	236
0.1mM	135	191	189	142
0.25mM	81	114	114	88
0.5mM	46	90	86	65
1mM	56	95	90	33
col-10p:tir1, raga-1-aid				
IAA	L1	L2	L3	L4
0mM	77	106	105	105
0.1mM	41	70	70	69
0.25mM	70	103	103	101
0.5mM	58	93	92	91
1mM	27	42	42	39
myo-2p:tir-1, daf-2-aid				
IAA	L1	L2	L3	L4
0mM	27	47	47	47
0.1mM	34	49	49	49
0.25mM	32	57	57	57
0.5mM	24	41	41	39
1mM	18	31	31	29
myo-2p:tir1, raga-1-aid, daf-16(mu86)				
IAA	L1	L2	L3	L4
0mM	105	152	151	134
0.1mM	154	198	195	140
0.25mM	64	89	86	71
0.5mM	32	51	51	42
1mM	41	71	71	38

col-10p:tir1, raga-1-aid, daf-16(mu86)				
IAA	L1	L2	L3	L4
0mM	34	61	61	59
0.1mM	23	40	40	39
0.25mM	39	57	57	57
0.5mM	25	41	41	40
1mM	36	45	45	44
no Tir1 expression, *raga-1-aid* , *daf-16(mu86)*				
IAA	L1	L2	L3	L4
0mM	50	102	101	84
0.1mM	48	77	77	57
no Tir expression, raga-1-aid, 0.5 mM IAA, mock RNAi				
hatch	M1	M2	M3	M4
39	72	71	71	66
no Tir expression, *raga-1-aid* , 0.5 mM IAA, *yap-1(RNAi)*				
hatch	M1	M2	M3	M4
62	71	70	70	70
col-10p:tir1 , *raga-1-aid* , 0.5 mM IAA, mock RNAi				
hatch	M1	M2	M3	M4
36	63	63	63	62
col-10p:tir1 , *raga-1-aid* , 0.5 mM IAA, *yap-1(RNAi)*				
65	98	80	59	31
RNAi screen (Fig. 5a,c)				
RNAi	no Tir	col-10:Tir		
EV	110	98		
dbl-1	33	34		
lon-1	17	13		
wts-1	13	16		
yap-1	71	98		
ftt-2	9	16		
rsks-1	27	24		
wild type (Fig. 5b,d,e-g,I,j)				
hatch	M1	M2	M3	M4
86	182	182	180	173
yap-1(tm1416) (Fig. 5b,d,e-g,I,j)				
hatch	M1	M2	M3	M4
178	363	375	374	370
col-10p:tir1 , *raga-1-aid* , 0.5 mM IAA (Fig. 5b,d,e-g,I,j)				
hatch	M1	M2	M3	M4
16	70	70	70	70
col-10p:tir1 , *raga-1-aid* , 0.5 mM IAA, *yap-1(tm1416)* (Fig. 5b,d,e-g,I,j)				
hatch	M1	M2	M3	M4
49	72	69	69	68

Supplemental Table 3. p-values for data in Figure 5

p-value for interaction term of 2-way ANOVA for interaction between epidermal RAGA-1 AID and yap-1(RNAi) or yap-1(tm1416)

	yap-1(RNAi)	yap-1(tm1416)
growth rate L4	1.61E-45	3.31E-20
L4 duration	1.37E-44	1.12E-19
body length at M4length	1.49E-15	5.98E-09
pharynx length at M4	1.42E-16	1.95E-23
deviation pharynx length from P-line M	3.13E-10	2.40E-22

p-values pariwise comparisons (ranksum test, two-sided)

body growth rate L4

	mock RNAi, no AID	yap-1(RNAi), no AID	mock RNAi, epidermal RAGA-1 AID	yap-1(RNAi), epidermal RAGA-1 AID
mock RNAi, no AID		1.19E-10	2.17E-34	3.44E-17
yap-1(RNAi), no AID			3.43E-28	1.40E-15
mock RNAi, epidermal RAGA-1 AID				3.86E-16
yap-1(RNAi), epidermal RAGA-1 AID				

L4 duration

	mock RNAi, no AID	yap-1(RNAi), no AID	mock RNAi, epidermal RAGA-1 AID	yap-1(RNAi), epidermal RAGA-1 AID
mock RNAi, no AID		0.146169018	3.37E-18	2.47E-17
yap-1(RNAi), no AID			2.02E-20	9.95E-16
mock RNAi, epidermal RAGA-1 AID				5.50E-16
yap-1(RNAi), epidermal RAGA-1 AID				

body length at M4

	mock RNAi, no AID	yap-1(RNAi), no AID	mock RNAi, epidermal RAGA-1 AID	yap-1(RNAi), epidermal RAGA-1 AID
mock RNAi, no AID		8.87E-07	8.52E-34	3.44E-17
yap-1(RNAi), no AID			4.41E-28	1.40E-15
mock RNAi, epidermal RAGA-1 AID				2.80E-16
yap-1(RNAi), epidermal RAGA-1 AID				

pharynx length at M4

	mock RNAi, no AID	yap-1(RNAi), no AID	mock RNAi, epidermal RAGA-1 AID	yap-1(RNAi), epidermal RAGA-1 AID
mock RNAi, no AID		6.42E-12	4.72E-10	3.39E-16
yap-1(RNAi), no AID			0.147566704	3.22E-15
mock RNAi, epidermal RAGA-1 AID				2.55E-16
yap-1(RNAi), epidermal RAGA-1 AID				

deviation pahrynx length from P-line

	mock RNAi, no AID	yap-1(RNAi), no AID	mock RNAi, epidermal RAGA-1 AID	yap-1(RNAi), epidermal RAGA-1 AID
mock RNAi, no AID		3.84E-08	2.49E-21	1.90E-05
yap-1(RNAi), no AID			2.48E-25	0.204036665
mock RNAi, epidermal RAGA-1 AID				6.87E-15
yap-1(RNAi), epidermal RAGA-1 AID				

body growth rate L4

	wild type, no AID	yap-1(tm1416), no AID	wild type, epidermal RAGA-1 AID	yap-1(tm1416), epidermal RAGA-1 AID
wild type, no AID		3.91E-19	4.66E-34	1.54E-33
yap-1(tm1416), no AID			3.31E-40	2.81E-39
wild type, epidermal RAGA-1 AID				5.33E-09
yap-1(tm1416), epidermal RAGA-1 AID				

L4 duration

	wild type, no AID	yap-1(tm1416), no AID	wild type, epidermal RAGA-1 AID	yap-1(tm1416), epidermal RAGA-1 AID
wild type, no AID		0.048375014	7.33E-25	1.95E-32
yap-1(tm1416), no AID			1.50E-36	9.33E-40
wild type, epidermal RAGA-1 AID				1.19E-12
yap-1(tm1416), epidermal RAGA-1 AID				

body length at M4

	wild type, no AID	yap-1(tm1416), no AID	wild type, epidermal RAGA-1 AID	yap-1(tm1416), epidermal RAGA-1 AID
wild type, no AID		1.76E-13	3.24E-33	3.86E-33
yap-1(tm1416), no AID			1.15E-39	6.77E-39
wild type, epidermal RAGA-1 AID				0.019718967
yap-1(tm1416), epidermal RAGA-1 AID				

pharynx length at M4

	wild type, no AID	yap-1(tm1416), no AID	wild type, epidermal RAGA-1 AID	yap-1(tm1416), epidermal RAGA-1 AID
wild type, no AID		0.003458426	1.21E-15	3.33E-33
yap-1(tm1416), no AID			5.97E-08	9.69E-36
wild type, epidermal RAGA-1 AID				3.85E-23
yap-1(tm1416), epidermal RAGA-1 AID				

deviation pharynx length from P-line

	wild type, no AID	yap-1(tm1416), no AID	wild type, epidermal RAGA-1 AID	yap-1(tm1416), epidermal RAGA-1 AID
wild type, no AID		8.29E-13	4.63E-05	2.93E-28

yap-1(tm1416), no AID			1.44E-18	2.45E-20
wild type, epidermal RAGA-1 AID				2.91E-22
yap-1(tm1416), epidermal RAGA-1 AID				

Supplemental Table 4. List of strains used in this study

wBT125	*bqSi577 [myo-2p::gfp] IV.; wbmIs88 [eft-3p::3xflag::dpy-10 crRNA::sl2::wrmscarlet::unc-54 3' UTR] V.*
wBT137	*ieSi60 [myo-2p::tir1::mruby::unc-54 3'UTR] II.; daf-2(bch40 [aid::3xflag::STOP::sl2-sv40::wrmscarlet-nls]) III; bqSi577 [myo-2p::gfp] IV.; wbmIs88 [eft-3p::3xflag::dpy-10 crRNA::sl2::wrmscarlet::unc-54 3' UTR] V.*
wBT160	*raga-1(wbm40 [raga-1::aid::gfp]) II.; bqSi577 [myo-2p::gfp] IV.; wbmIs88 [eft-3p::3xflag::dpy-10 crRNA::sl2::wrmscarlet::unc-54 3' UTR] V.*
wBT182	*raga-1(wbm40 [raga-1::aid::gfp]) ieSi60[myo-2p::tir1::mruby::unc-54 3'UTR] II.; bqSi577 [myo-2p::gfp] IV.; wbmIs88 [eft-3p::3xflag::dpy-10 crRNA::sl2::wrmscarlet::unc-54 3' UTR] V.*
wBT186	*reSi1 [col-10p::tir1::f2a::mtagbfp2::nls::aid::tbb-2 3'UTR] I.; raga-1(wbm40 [raga-1::aid::gfp]) II.;bqSi577 [myo-2p::gfp] IV.; wbmIs88 [eft-3p::3xflag::dpy-10 crRNA::sl2::wrmscarlet::unc-54 3' UTR] V.*
wBT190	*raga-1(wbm40 [raga-1::aid::gfp]) II.; xeSi376 [eft-3p::tir1::mruby::unc-54 3'UTR] III.; bqSi577 [myo-2p::gfp] IV.; wbmIs88 [eft-3p::3xflag::dpy-10 crRNA::sl2::wrmscarlet::unc-54 3' UTR] V.*
wBT258	*daf-16(mu86) I.; raga-1(wbm40 [raga-1::aid::gfp]) ieSi60 [myo-2p::tir1::mruby::unc-54 3'UTR] II.; bqSi577 [myo-2p::gfp] IV.; wbmIs88 [eft-3p::3xflag::dpy-10 crRNA::sl2::wrmscarlet::unc-54 3' UTR] V.*
wBT263	*daf-16(mu86) I. raga-1(wbm40 [raga-1::aid::gfp]) II.; bqSi577 [myo-2p::gfp] IV.; wbmIs88 [eft-3p::3xflag::dpy-10 crRNA::sl2::wrmscarlet::unc-54 3' UTR] V.*
wBT264	*reSi1 [col-10p::tir1::f2a::mtagbfp2::nls::aid::tbb-2 3'UTR] daf-16(mu86) I.; raga-1(wbm40 [raga-1::aid::gfp]) II.;bqSi577 [myo-2p::gfp] IV.; wbmIs88 [eft-3p::3xflag::dpy-10 crRNA::sl2::wrmscarlet::unc-54 3' UTR] V.*
wBT310	*bqSi577 [myo-2p::GFP + unc-119(+)] IV; wbmIs88 [eft-3p::3xFLAG::dpy-10 crRNA::SL2::wrmscarlet::unc-54 3' UTR] V:8645000; yap-1(tm1416) X*
wBT337	*reSi1 [col-10p::TIR1::F2A::mTagBFP2::NLS::AID::tbb-2 3'UTR] (I:-5.32); raga-1(wbm40) [raga-1::AID::EmGFP] II; bqSi577 [myo-2p::GFP + unc-119(+)] IV; wbmIs88 [eft-3p::3xFLAG::dpy-10 crRNA::SL2::wrmscarlet::unc-54 3' UTR] V; yap-1(tm1416) X*
